# Thermal intervention improves pulse oximetry accuracy in critically ill patients with low perfusion: a quasi-experimental study

**DOI:** 10.1186/s40560-026-00847-w

**Published:** 2026-01-09

**Authors:** Natalie Karlsson, Felicia Olsson Lindstrand, Lotta Johansson, Carl Sjödin

**Affiliations:** 1 Department of Thoracic Anaesthesiology and Intensive Care, Region Västra Götaland, Sahlgrenska University Hospital, Gothenburg, Sweden; 2 Department of Anaesthesiology and Intensive Care, Region Västra Götaland, Sahlgrenska University Hospital, Gothenburg, Sweden; 3 Institute of Health and Care Sciences at the Sahlgrenska Academy, University of Gothenburg, Gothenburg, Sweden

**Keywords:** Intensive care, Pulse oximetry, Perfusion index, Microcirculation, Oxygen monitoring, Patient safety

## Abstract

**Background:**

Pulse oximetry is essential for continuous oxygen monitoring in intensive care, yet its accuracy declines in patients with low peripheral perfusion, risking both unrecognised hypoxaemia and inappropriate oxygen therapy. The perfusion index (PFI) reflects peripheral blood flow and is often reduced in critically ill patients with impaired microcirculation. Simple bedside strategies to restore PFI and improve SpO₂ accuracy remain underexplored.

**Methods:**

In this prospective quasi-experimental study, 46 adult ICU patients with arterial catheters and baseline PFI < 1.0 underwent localised peripheral warming using a wrist–forearm heating pad for 15 min. The warming pad maintained a surface temperature of 54 °C, with directly measured skin-interface temperatures of 41–42 °C. SpO₂, PFI, and arterial oxygen saturation (SaO₂) were measured immediately before and after the intervention. The primary outcome was the change in PFI; the secondary outcome was the improvement in SpO₂ accuracy (SpO₂–SaO₂ bias).

**Results:**

Thermal intervention increased PFI from a median (IQR) of 0.56 (0.34–0.78) to 3.59 (2.45–4.77) (*p* < 0.001; Hedges’ *g* = 2.43). The pre-intervention SpO₂–SaO₂ bias was 4.09% (95% limits of agreement 0.61–7.56%), which decreased to 0.00% (− 1.22–1.23%) after warming. Improvements were consistent across subgroups and unrelated to cardiac index, vasoactive use, or skin pigmentation.

**Conclusions:**

A brief, localised thermal intervention markedly improves peripheral perfusion and restores pulse-oximetry accuracy to within the clinically acceptable ± 2% range in critically ill patients with low PFI. However, ceiling effects at SpO₂ values near 100% and the pre–post design limit the strength of causal inference. This simple, non-invasive technique can be readily integrated into ICU practice to enhance the reliability of oxygen monitoring and reduce the risk of undetected hypoxaemia or hyperoxaemia.

**Supplementary Information:**

The online version contains supplementary material available at 10.1186/s40560-026-00847-w.

## Introduction

Pulse oximetry is a cornerstone of non-invasive monitoring in intensive care, providing continuous estimates of arterial oxygen saturation (SpO₂) along with perfusion index (PFI), heart rate, and waveform quality [1]. SpO₂ is calculated by analysing red and infrared light absorption through peripheral tissue, while PFI, derived from the same waveform, reflects the ratio of pulsatile to non-pulsatile blood flow and serves as an indirect measure of peripheral circulation and vasomotor tone [2, 3].

Although widely used, pulse oximetry has recognised limitations. Most accuracy data come from healthy volunteers, limiting applicability to critically ill patients [4, 5]. Accuracy is reduced by factors such as skin pigmentation, motion artefacts, temperature, nail polish, and critically—low peripheral perfusion [1, 5–9]. Under such conditions, SpO₂ often overestimates arterial oxygen saturation (SaO₂), potentially leading to undetected hypoxaemia [6, 10].

Low PFI and prolonged capillary refill time (CRT) are not only markers of reduced peripheral perfusion but have also been associated with impaired microcirculatory oxygen delivery and increased mortality in several ICU populations [11–17]. Patients with these signs may be particularly vulnerable to the consequences of undetected hypoxaemia, making accurate and reliable pulse oximetry essential for timely intervention.

Low PFI values are typically seen with vasoconstriction, hypovolaemia, or low cardiac output [3, 18]. Manufacturer guidelines suggest PFI > 1.0% is optimal, with < 0.3% considered marginal, but no consensus threshold exists [19]. Studies have shown SpO₂ readings become less reliable as PFI falls [2, 6], yet few have tested interventions aimed at improving PFI and reducing SpO₂–SaO₂ discrepancies in ICU patients.

Thermal stimulation is a plausible approach: local warming induces vasodilation, potentially improving peripheral perfusion and sensor signal quality [20, 21]. While proposed as a strategy to enhance pulse oximetry [20], supporting evidence in critically ill patients is lacking.

This study aimed to determine whether peripheral warming increases PFI and improves SpO₂ accuracy in critically ill patients with low baseline PFI. Secondary objectives were to assess whether PFI changes correlated with improved SpO₂ accuracy and whether clinical variables, such as haemodynamic, vasoactive use, or skin tone, modified the effect of thermal intervention. No previous ICU studies have systematically evaluated targeted thermal intervention as a means to restore pulse-oximetry accuracy during low peripheral perfusion.

## Methods

### Study design and ethics

This prospective quasi-experimental pre–post study was conducted between January and September 2024 in three ICUs at Sahlgrenska University Hospital, Gothenburg, Sweden: the central intensive care unit (CIVA; 1,797 admissions in 2024), the neuro-ICU (NIVA; 403 admissions in 2024), and the thoracic ICU (TIVA; 866 admissions in 2024), according to the Swedish Intensive Care Registry (SIR). Each patient served as their own control, minimising inter-individual variability in perfusion and skin temperature.

The study was approved by the Swedish Ethical Review Authority (Dnr: 2023-06496-01) and conducted in accordance with the Declaration of Helsinki. Written informed consent was obtained from all patients or, if the patient was unable to consent, from next of kin in accordance with presumed patient wishes.

### Clinical trial registration

Not applicable. This was a non-randomised, quasi-experimental investigation, with objectives and methodology fully described in the approved ethics application. It did not meet the ICMJE criteria for mandatory prospective trial registration [22].

### Participants

Inclusion criteria:

- Aged ≥ 18 years
- Arterial catheter in situ
- Continuous pulse oximetry with a Masimo RADICAL™ RD-4050 connected to a Philips MX800 monitor
- Baseline PFI < 1.0 (manufacturer threshold for low perfusion; associated with reduced SpO₂ accuracy in ICU[19]).

Exclusion criteria:

- Nail polish or acrylic nails
- Extracorporeal life support (ECLS)
- Durable or temporary LVAD
- Intra-aortic balloon pump (IABP)
- Absent or unstable pulse oximetry signal (no detectable PFI waveform).

### Intervention

The intervention was performed during a stable phase of patient care with no concurrent procedures or treatment changes. All measurements were conducted during normoxic or near-normoxic conditions, as maintaining prolonged desaturation during the intervention was considered unethical.

Local warming was performed using a LectroDerm™ pad (19 × 29 cm; Handelshuset Viroderm, Sweden) with thermostatic control maintaining a surface temperature of 54 °C. A single-use protective cover was applied, and the temperature beneath the cover, at the skin interface, was directly measured at 41–42 °C throughout the procedure, consistent with previous microvascular warming protocols. The pad was positioned over the wrist and forearm for 15 min.

The pulse-oximeter sensor was placed on the same index, middle, or ring finger throughout the procedure, with signal quality verified prior to data collection.

In addition to finger PFI, SpO₂, and SaO₂, PFI was also measured from the earlobe before and after the intervention using a dedicated Masimo RD earlobe sensor connected to the same Philips MX800 bedside monitor. The earlobe site was not subjected to warming and served as a control to assess whether changes in PFI were due to local thermal effects rather than systemic haemodynamic alterations.

### Data collection

All measurements were obtained during a clinically stable phase of care, with no procedural or treatment changes between time points. Local warming was performed using a LectroDerm™ pad (19 × 29 cm; Handelshuset Viroderm, Sweden) with thermostatic control maintaining a surface temperature of 54 °C. A single-use protective cover was applied. The temperature at the skin–pad interface was continuously measured using a disposable esophageal/rectal temperature probe (Datex-Ohmeda) placed beneath the protective cover directly against the skin. The measured interface temperature remained 41–42 °C throughout the intervention, consistent with prior microcirculatory warming protocols in critically ill patients [20, 23]. The pad was positioned over the wrist and forearm for 15 min.

Arterial blood gases (SaO₂) were drawn immediately before and after the 15-min warming period. To ensure temporal alignment between SaO₂ and SpO₂ measurements, the SpO₂ value used for analysis was recorded within approximately ± 15 s of each arterial blood gas sampling time.

The pulse-oximeter sensor remained on the same finger throughout to eliminate positional variability, and signal quality was verified continuously. Earlobe PFI was recorded immediately before and after the intervention using a dedicated Masimo RD earlobe sensor that was not warmed, serving as an internal control to distinguish local from systemic effects.

Baseline demographic, clinical, and physiological variables, including cardiac index, body temperature, arterial pressure, vasoactive agent use, and skin pigmentation, were collected concurrently. Skin pigmentation was classified according to the Fitzpatrick scale [24].

Throughout the procedure, the skin at the warmed site was inspected before and after warming, and ICU nurses monitored skin integrity continuously to ensure patient safety, particularly because many critically ill patients are unable to report thermal discomfort.

### Statistical analysis

A priori sample size estimation was based on pilot data from 20 spontaneously breathing ICU and postoperative patients showing a mean PFI increase ≈ 3 (± 1 SD) after warming. Assuming *α* = 0.05 (two-sided) and 80% power to detect a 2-unit PFI increase (considered clinically relevant for signal recovery), minimum *n* = 20. To improve precision and allow for attrition and non-normal distribution, 46 patients were included. This sample size was therefore sufficient to detect large within-subject changes in PFI but not powered to test secondary associations (e.g., ΔPFI vs ΔSpO₂–SaO₂ difference). Accordingly, these exploratory analyses are interpreted descriptively rather than inferentially.

Normality of continuous data was assessed visually and using the Shapiro–Wilk test. Continuous variables are presented as mean ± standard deviation (SD) if normally distributed, or as median and interquartile range (IQR) if skewed. PFI values demonstrated a skewed distribution; therefore, changes in PFI were analysed using the Wilcoxon signed-rank test. Agreement between SpO₂ and SaO₂ was evaluated using Bland–Altman analysis (bias and 95% limits of agreement) [25], and systematic differences were tested using the Wilcoxon signed-rank test. Associations between PFI change and clinical variables were examined using Spearman’s correlation coefficient.

## Results

### Study population

**Fig. 1:**
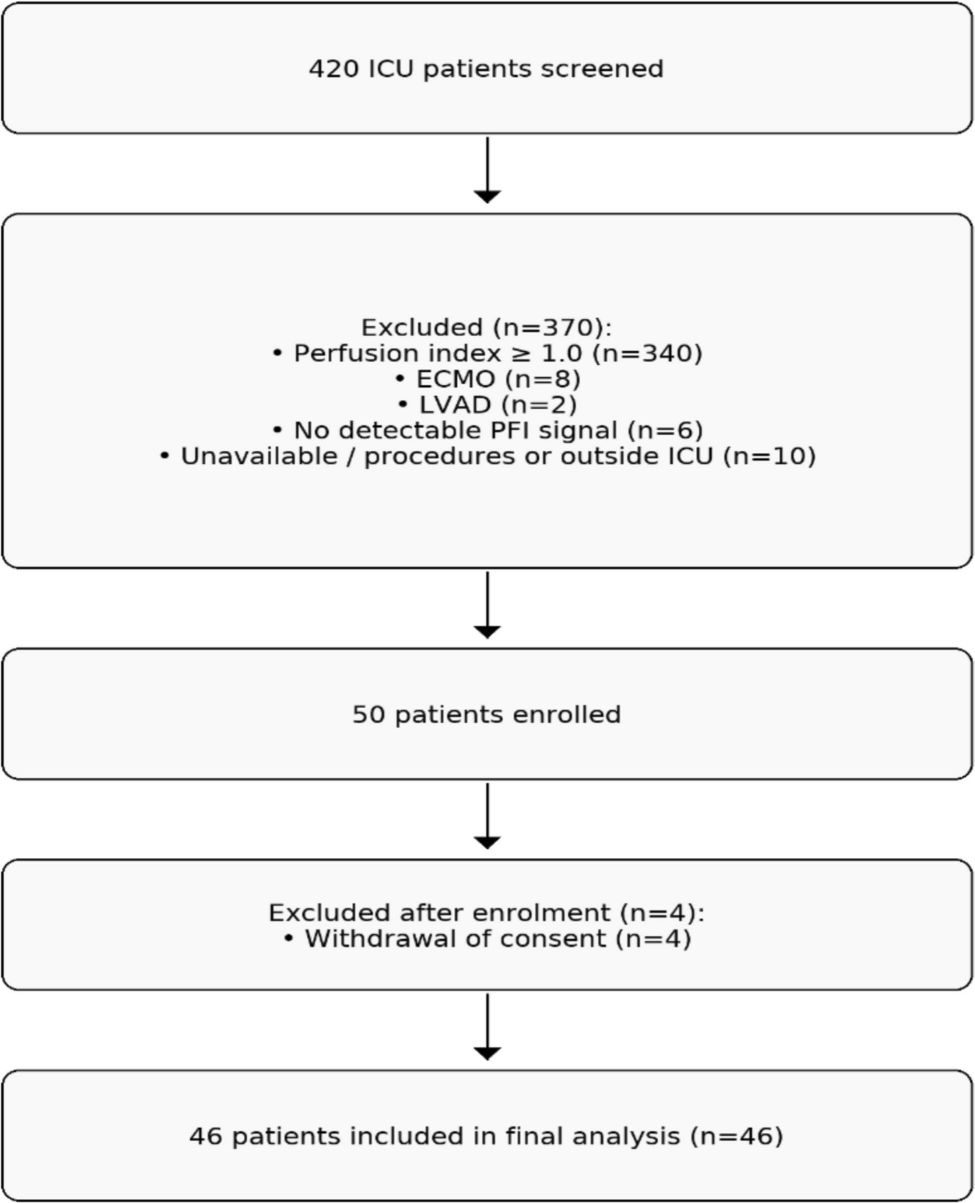
Patient screening and inclusion flowchart. Of 420 ICU patients screened, 50 met inclusion criteria and were enrolled. A total of 370 were excluded due to: perfusion index ≥ 1.0 (*n* = 340), extracorporeal membrane oxygenation (ECMO, *n* = 8), left ventricular assist device (LVAD, *n* = 2), no detectable perfusion index signal (*n* = 6), or unavailability due to concurrent procedures or care outside the ICU (*n* = 10)

Between January and September 2024, a total of 420 ICU patients were screened for eligibility. Of these, 340 (81%) had a perfusion index (PFI) ≥ 1.0 and were excluded, while 66 (15.7%) exhibited low or absent peripheral perfusion (PFI < 1.0 or no detectable signal). Among this subgroup, 10 patients were unavailable for inclusion due to concurrent procedures or care outside the ICU, and 6 were excluded because no PFI signal could be detected. The remaining 50 patients were enrolled. Four subsequently withdrew consent, leaving 46 patients for final analysis (Fig. 1).

Baseline characteristics are summarised in Table 1. The mean ± SD age was 57 ± 14 years, and 36% were female. The median (IQR) baseline PFI was 0.40 (0.20–0.60), consistent with markedly reduced peripheral perfusion. A substantial proportion of patients were receiving vasopressor therapy at inclusion (55%), and 66% were mechanically ventilated. Illness severity was high, reflected by a median (IQR) SOFA score of 12 (9–15). Despite low peripheral perfusion, systemic haemodynamics were generally stable, with a mean arterial pressure of 73 ± 8 mmHg and a mean (± SD) core temperature of 37.0 ± 0.75 °C. Among the 16 patients with continuous cardiac output monitoring, the mean cardiac index was 3.0 ± 0.69 L/min/m^2^.

**Table 1:** Baseline characteristics and haemodynamics before and after thermal intervention (*n* = 46)

Variable	Pre	Post
FPS, *n* (%)	Type 1: 13 (28.3); Type 2: 25 (54.3); Type 3: 4 (8.7); Type 4: 3 (6.5); Type 5: 1 (2.2)	–
Admission diagnosis, *n* (%)	Surgical 52; Shock/Sepsis 26; Neuro 22	–
Mechanical ventilation, *n* (%)	30 (65.3)	–
SOFA score, median (IQR)	12 (9–15)	–
Weight (kg), mean ± SD	81.0 ± 17.3	–
Temperature (°C), mean ± SD	37.0 ± 0.75	37.0 ± 0.77
Perfusion index (PFI), median (IQR)	0.55 (0.34–0.78)	3.59 (2.45–4.77)
SpO₂ (%), mean ± SD	100 ± 0.42	96 ± 1.79
SaO₂ (%), mean ± SD	96 ± 1.85	96 ± 1.78
pH (ABG), mean ± SD	7.40 ± 0.05	7.40 ± 0.05
Haemoglobin (g/dL), mean ± SD	108.9 ± 15.1	109 ± 15.0
SBP (mmHg), mean ± SD	99 ± 13.9	100 ± 13.8
DBP (mmHg), mean ± SD	59 ± 7.9	60 ± 7.7
MAP (mmHg), mean ± SD	73 ± 8.43	75 ± 9.26
Propofol (mg/kg/h), median (IQR)	1.0 (0.5–1.4)	1.0 (0.5–1.4)
Norepinephrine (µg/kg/min), median (IQR)	0.15 (0.0–0.24)	0.15 (0.0–0.24)
Vasopressin (mL/h), median (IQR)	0.32 (0.0–1.05)	0.32 (0.0–1.05)
Continuous CO monitoring, *n* (%)	16 (35)	–
Cardiac index (L/min/m^2^), mean ± SD (*n* = 16)	3.0 ± 0.69	3.0 ± 0.75

Abbreviations: FPS = Fitzpatrick skin phototype score; ABG = arterial blood gas; SBP = systolic blood pressure; DBP = diastolic blood pressure; MAP = mean arterial pressure; SOFA = Sequential Organ Failure Assessment

After enrolment, 4 patients withdrew consent, leaving 46 patients included in the final analysis.

PFI = perfusion index; ECMO = extracorporeal membrane oxygenation; LVAD = left ventricular assist device.

### Perfusion index changes after thermal intervention

**Fig. 2:**
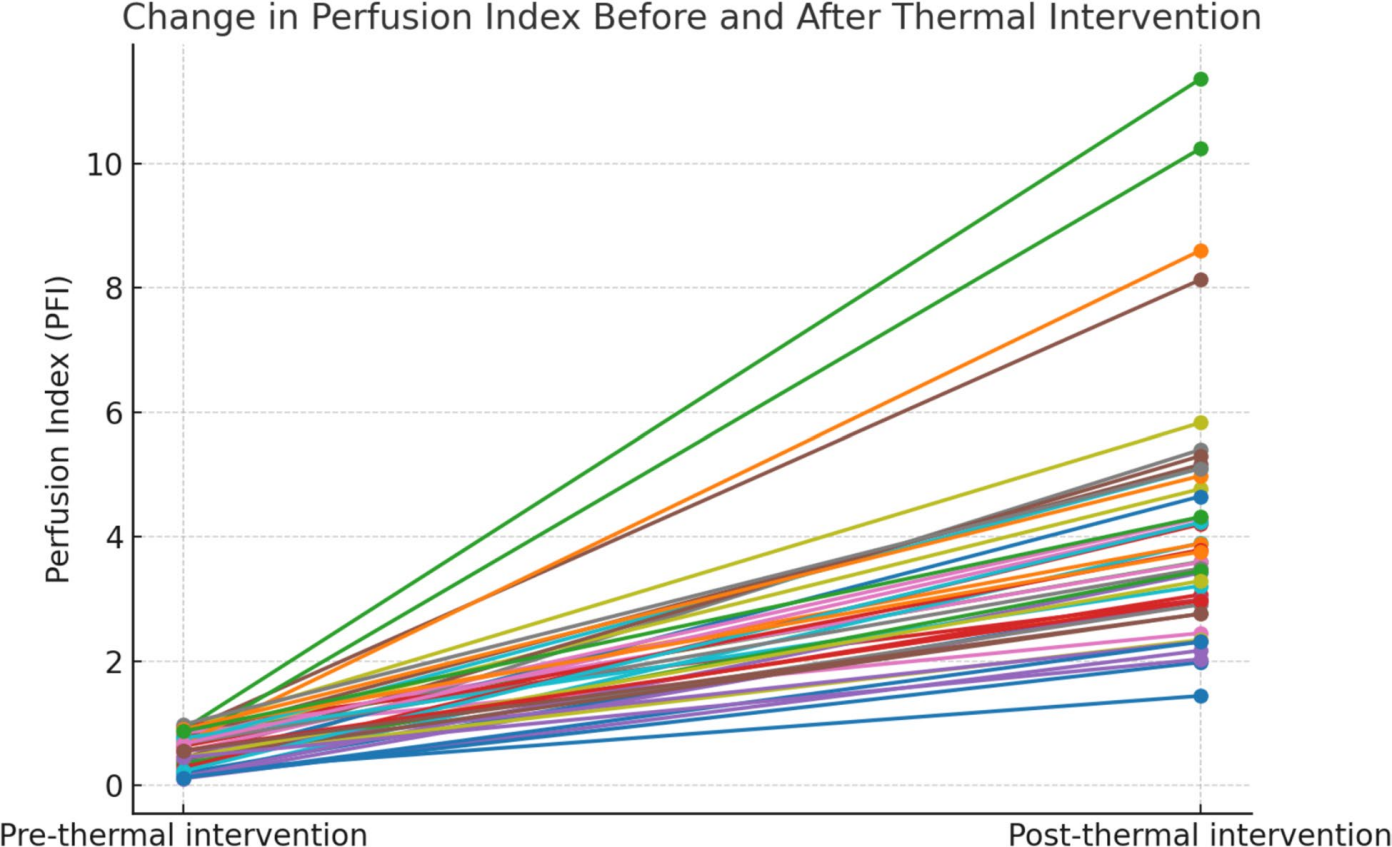
Individual changes in perfusion index (PFI) before and after a 15-min local thermal intervention in 46 critically ill ICU patients with baseline PFI < 1.0. Each line represents one patient. PFI increased in all participants following thermal intervention

PFI increased significantly after the thermal intervention from a median (IQR) of 0.56 (0.34–0.78) to 3.59 (2.45–4.77) (*p* < 0.001, Wilcoxon signed-rank test) (Fig. 2). The effect size was large (Hedges’ *g* = 2.43). Pre- and post-intervention PFI values showed a strong correlation (*R*^*2*^ = 0.987), indicating a consistent improvement in peripheral perfusion across patients. PFI measured at the earlobe, used as a control site not subjected to warming, showed no significant change pre- and post-intervention (median [IQR] 0.79 [0.49–1.20] vs. 0.81 [0.56–1.20], p = 0.131) (Supplementary file, Fig. S2).

### Effect of thermal intervention on SpO₂ accuracy

**Fig. 3:**
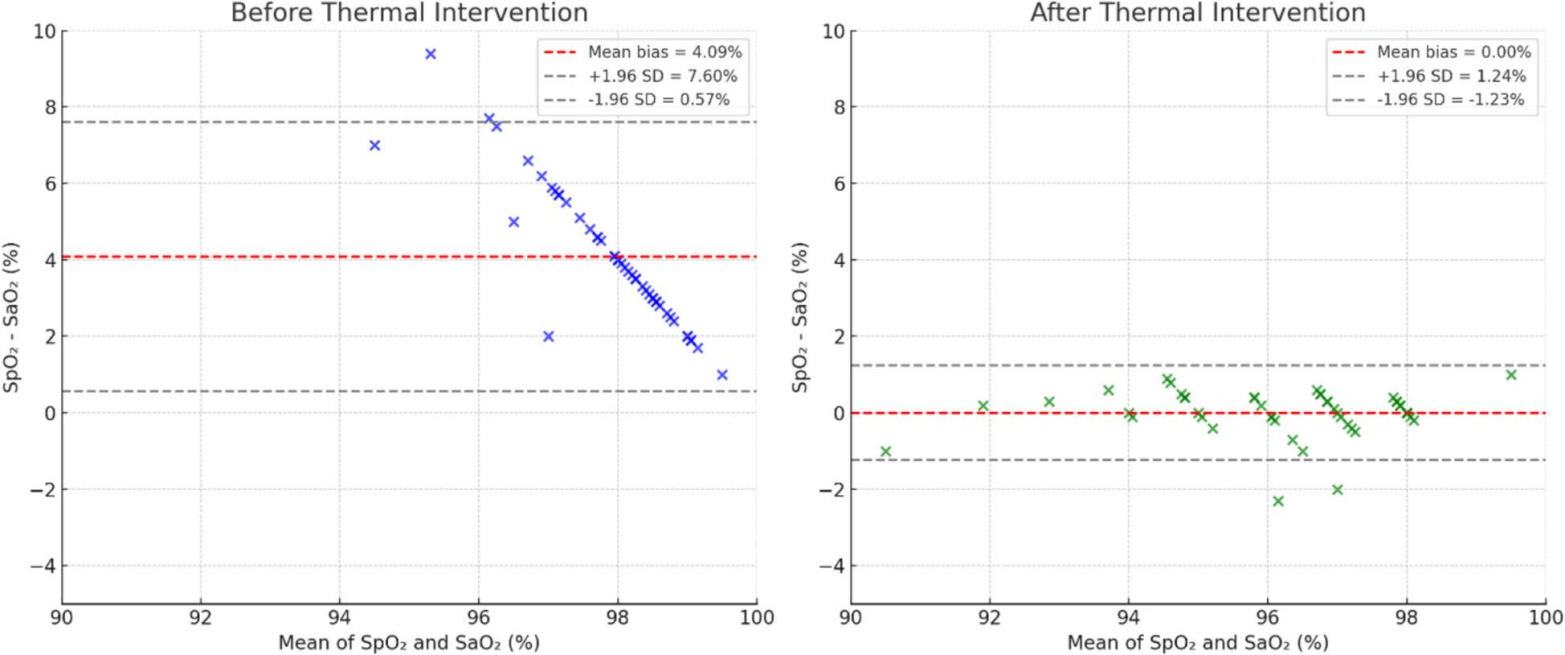
Bland–Altman plots showing the difference between arterial oxygen saturation (SaO₂) and pulse oximetry (SpO₂) before (left) and after (right) thermal intervention. The solid horizontal line indicates mean bias, and the dashed lines represent the 95% limits of agreement

Before thermal intervention, SpO₂ overestimated SaO₂ by a mean bias of 4.09%, with 95% limits of agreement ranging from 0.61% to 7.56% (Fig. 3). After thermal intervention, the bias decreased to 0.00%, and the limits of agreement narrowed markedly to − 1.22% to 1.23% (Fig. 3). The pre-intervention bias was statistically significant (*p* < 0.001), whereas the post-intervention bias was not (*p* = 0.963).

There was no statistically significant correlation between ΔPFI and the reduction in SpO₂–SaO₂ discrepancy (Spearman’s *ρ* = 0.25, *p* = 0.09) (Supplementary file, Fig. S4). This indicates that although thermal intervention improved both peripheral perfusion and pulse oximetry accuracy, the magnitude of perfusion improvement was not directly proportional to the reduction in SpO₂ bias.

### Physiological and clinical factors associated with PFI change

Correlation analyses were performed to explore factors associated with the change in finger PFI after thermal intervention (Table 2). None of the examined variables showed a statistically significant correlation with ΔPFI. The correlations were weak for changes in cardiac index (*ρ* = 0.05, *p* = 0.79), mean arterial pressure (*ρ* = 0.046, *p* = 0.76), body temperature (*ρ* = 0.01, *p* = 0.93), baseline norepinephrine dose (*ρ* =  − 0.21, *p* = 0.16), and Fitzpatrick skin phototype score (*ρ* =  − 0.11, *p* = 0.46).

**Table 2:** Spearman’s rank correlation between change in perfusion index (ΔPFI) and selected clinical variables. *N* = 46

Variable	*ρ*	*p*-value
ΔCardiac index (*n* = 16)	0.05	0.79
ΔMean arterial pressure	0.046	0.76
ΔBody temperature	0.01	0.93
Baseline norepinephrine dose	− 0.21	0.16
Fitzpatrick skin phototype	− 0.11	0.46

Δ indicates post-intervention minus pre-intervention values. Correlation coefficients (*ρ*) with corresponding *p*-values are shown. *p* < 0.05 considered statistically significant; none of the correlations reached statistical significance.

### Safety

No adverse events were observed during or after the thermal intervention. Skin inspection showed no erythema or burns.

## Discussion

This study demonstrates that a brief, local thermal intervention substantially improves peripheral perfusion and the accuracy of pulse oximetry in critically ill patients with low baseline PFI. Thermal intervention increased PFI from a median (IQR) of 0.56 (0.34–0.78) to 3.59 (2.45–4.77), representing a large effect size (Hedges’ *g* = 2.43). The SpO₂–SaO₂ bias decreased from 4.09% before thermal intervention to 0.00% after, with 95% limits of agreement narrowing from 0.61 to 7.56% pre-intervention to − 1.22–1.23% post-intervention. These changes brought measurement accuracy well within the clinically acceptable ± 2% range [26], suggesting a meaningful improvement in reliability for bedside monitoring.

Although the within-patient pre–post design reduces inter-individual variability, it remains vulnerable to unmeasured temporal fluctuations in haemodynamics. Even with stable systemic variables and unchanged earlobe PFI, small changes in vasomotor tone or microcirculatory flow cannot be fully excluded. Therefore, the findings should be interpreted as demonstrating physiological plausibility rather than definitive causal confirmation of the effect of thermal intervention on SpO₂ accuracy.

Interpretation of the pre-intervention bias is partly limited by ceiling effects inherent to pulse oximetry at SpO₂ values near 100%, where device algorithms truncate displayed values and reduce visible variability. When arterial saturations are slightly below 100%, the oximeter may still display a ceiling value, artificially inflating the apparent SpO₂–SaO₂ discrepancy. Similar saturation-plateau effects have been described in prior pulse-oximetry accuracy studies, particularly in critically ill patients and during low-perfusion states [4, 5, 27].

Interestingly, the magnitude of PFI improvement was only weakly correlated with the reduction in SpO₂–SaO₂ bias (*ρ* = 0.25, *p* = 0.09). The absence of a statistically significant association between ΔPFI and ΔSpO₂–SaO₂ discrepancy suggests that the mechanism by which warming restores accuracy may not be directly proportional to the magnitude of perfusion change. It is also possible that the study was underpowered to detect this relationship, as the sample size was calculated for the primary endpoint (PFI change) rather than for secondary associations. Beyond statistical considerations, accuracy improvements may reflect crossing a critical perfusion threshold beyond which the signal-to-noise ratio improves disproportionately [2, 27, 28], or temperature-mediated reductions in photoplethysmographic artefact independent of absolute PFI change. These alternative mechanisms should be explored in future studies.

Importantly, these improvements occurred without significant changes in cardiac index, mean arterial pressure, norepinephrine dose, or body temperature, and were not influenced by skin pigmentation. Although factors such as acidosis, oedema, or oxygen saturation can affect the photoplethysmographic signal [29], these variables remained clinically stable during the measurement period, and each patient served as their own control. The absence of systemic haemodynamic effects, together with unchanged earlobe PFI, supports a local vasodilatory mechanism rather than global circulatory or metabolic changes as the most plausible explanation for the observed benefit.

This is clinically important because low PFI often persists despite adequate systemic perfusion pressure, particularly in patients on vasopressors [16, 30]. Importantly, the improvement in signal quality was achieved in less than 15 min, using a low-cost, widely available warming pad, without any adverse events. Although no adverse events occurred, critically ill patients often have impaired sensation, altered thermoregulation, or fragile skin that may increase vulnerability to thermal injury. Prior ICU studies using controlled local thermal challenges have safely applied temperatures up to 43 °C for microcirculatory assessment without harm, including in vasopressor-dependent patients [23]. Continuous inspection of the skin was performed during our intervention, but further evaluation in larger cohorts is needed to confirm safety in non–self-reporting or haemodynamically unstable patients. This makes the intervention highly feasible for integration into standard ICU nursing protocols, especially in patients with cold extremities, poor waveform quality, or unexplained SpO₂–SaO₂ discrepancies. However, the duration of this improvement beyond the intervention period was not evaluated, and whether repeated or prolonged warming could sustain enhanced perfusion and measurement reliability over time remains to be determined. Future studies should quantify how long the perfusion improvement persists, assess the decay time, and evaluate whether repeated or protocolised warming can maintain accurate SpO₂ measurements over longer observation periods.

The frequency of poor or absent pulse oximetry signals in critically ill patients further underscores the clinical relevance of these findings. In the present study, approximately 16% of screened ICU patients had low or absent PFI (< 1.0 or undetectable), and 1.5% had no measurable signal, values that align closely with prior reports indicating that 10–20% of ICU patients exhibit unreliable or low-quality SpO₂ signals due to vasoconstriction, hypoperfusion, or motion artefact, while < 5% experience complete signal dropout under severe circulatory compromise [1, 31, 32]. This concordance supports the representativeness of our sample and highlights that the studied population reflects a clinically relevant subset of ICU patients. In these patients, pulse oximetry, the cornerstone of continuous oxygenation monitoring, often fails precisely when accurate assessment is most critical. The observed ability of brief peripheral warming to restore strong, reliable signals therefore addresses a major gap in routine ICU monitoring and offers a practical, bedside intervention to enhance patient safety.

These findings have direct relevance for ICU practice. Low PFI reflects impaired peripheral microcirculation, which, together with prolonged capillary refill time, has been linked to reduced tissue oxygen delivery and increased mortality in multiple ICU populations [11, 13, 14, 16, 17]. In such patients, inaccurate SpO₂ readings risk delaying recognition of hypoxaemia or prompting unnecessary oxygen supplementation. Both scenarios can have harmful consequences: hypoxaemia through inadequate oxygen delivery to vital organs [33], and hyperoxaemia through oxidative injury and increased mortality in some patient groups [34–36]. By improving the reliability of SpO₂ measurements, peripheral warming provides a simple bedside strategy to reduce the risk of unrecognised hypoxaemia or hyperoxaemia.

Our results extend earlier physiological studies in healthy volunteers and non-ICU patients by confirming that thermal intervention can improve photoplethysmography signal quality and measurement accuracy in a critically ill population. Kamshilin et al. and Vallée et al. previously demonstrated that skin warming increases PFI and enhances microvascular gas exchange [20, 21]; our findings provide the first ICU-specific evidence linking these changes to a tangible improvement in oxygen saturation measurement accuracy. Unlike prior volunteer studies, this work controlled for systemic haemodynamic changes and used within-patient comparison, strengthening causal inference despite the absence of a randomised control group.

### Clinical implications

Improved SpO₂ reliability is particularly valuable in situations where arterial blood gas analysis is not immediately available, such as during rapid clinical deterioration, intrahospital transport, or in resource-limited ICU settings. Because most ICU protocols target SpO₂ values between 94 and 98%, even small systematic deviations can lead to clinically important misclassification of oxygenation status. Applying local peripheral warming in patients with a low perfusion index may therefore support safer oxygen titration and more accurate recognition of both hypoxaemia and hyperoxaemia. The unchanged cardiac index, mean arterial pressure, and earlobe PFI confirm that the observed improvement in fingertip PFI resulted from a local microvascular effect of warming rather than systemic haemodynamic changes, strengthening the physiological plausibility of this mechanism. Moreover, because unreliable oximetry signals remain common in everyday ICU practice, enabling nurses to apply simple, non-invasive interventions to restore reliable monitoring could meaningfully enhance patient safety and situational awareness in critical care.

### Limitations

This study has several limitations. The sample size was modest, though adequately powered for the primary endpoint, and the proportion of patients with complete advanced haemodynamic monitoring was limited. Only patients with detectable fingertip signals were included; therefore, generalisability to those with absent or unstable peripheral signals is uncertain. The cohort included relatively few participants with darker skin pigmentation, restricting conclusions about potential skin-tone effects. The pre–post design limits firm causal inference, as unmeasured temporal changes in haemodynamics cannot be entirely excluded, despite stable systemic variables and unchanged earlobe PFI.

Only patients with a detectable, albeit unreliable, pulse oximetry signal (low PFI) were included, representing approximately 10–20% of ICU patients who experience low perfusion but maintain measurable waveforms. In contrast, less than 5% of critically ill patients have no detectable signal due to profound vasoconstriction or circulatory collapse [1, 31, 32]. These patients were excluded, as reliable SpO₂ readings cannot be obtained under such conditions. Nevertheless, the prevalence of excluded cases because of no PFI signal (1.6% in this study) aligns with prior ICU data, supporting the representativeness of our sample. Interpretation of pre-intervention SpO₂–SaO₂ bias is also limited by ceiling effects at SpO₂ values near 100%, where device algorithms truncate displayed values and may artificially inflate apparent discrepancies.

The durability of the perfusion improvement beyond the 15-min intervention was not evaluated and should be addressed in longitudinal or repeated-intervention studies. Because the intervention required 15 min of stable monitoring, patients with SaO₂ values below clinically acceptable thresholds were not included for ethical reasons. Consequently, the findings primarily reflect conditions of reduced peripheral perfusion at normoxic or near-normoxic oxygen levels. However, since low PFI has consistently been associated with greater SpO₂–SaO₂ discrepancies in both experimental and clinical studies [2, 27, 28, 37, 38], it is plausible that similar or even stronger effects of thermal intervention could be observed in patients with lower arterial oxygen saturations. Future studies should examine this relationship under controlled and ethically acceptable conditions.

Safety considerations also apply: although skin–interface temperatures of 41–42 °C are consistent with prior ICU thermal-challenge protocols and no adverse events occurred, critically ill patients may have impaired sensation, altered thermoregulation, or fragile skin. Continuous monitoring of the warmed area is essential, and safety in non-responsive or vasopressor-dependent patients requires confirmation in larger cohorts.

Future studies should examine whether similar effects occur at lower oxygen saturations. Because each patient served as their own control under stable systemic conditions, the design isolates the local effect of warming but does not capture downstream clinical outcomes such as oxygen titration accuracy, hypoxaemia detection time, or incidence of hyperoxaemia. Finally, although the intervention clearly improved SpO₂ measurement reliability, the study was not powered nor designed to test whether this translates into better patient outcomes, an important goal for future multicentre trials.

### Generalisability

Although our cohort comprised critically ill ICU patients, the underlying physiological mechanism, local warming improving peripheral perfusion and pulse oximetry signal quality, is likely not limited to this setting. Perioperative patients frequently experience low peripheral perfusion due to hypothermia, vasopressor use, or sympathetic activation, conditions in which similar benefits might be observed. However, differences in anaesthesia, active warming strategies, and monitoring environments mean these results should be extrapolated with caution. Prospective evaluation in surgical populations is warranted to confirm applicability.

## Conclusion

Peripheral thermal stimulation is a simple, rapid, and inexpensive bedside intervention that significantly improves peripheral perfusion and enhances the reliability of pulse oximetry measurements in critically ill patients with low perfusion index. By reducing SpO₂ measurement bias, it may help prevent unrecognised hypoxaemia or hyperoxaemia and support safer, more precise oxygen titration in everyday ICU practice. Future multicentre studies should evaluate the sustainability, safety, and clinical impact of this approach, particularly whether improved monitoring reliability translates into better patient-centred outcomes across diverse ICU settings.

## Supplementary Information

Additional file1 (DOCX 468 kb)

## Data Availability

The data that support the findings of this study are available from Sahlgrenska University Hospital, Gothenburg. However, restrictions apply to the availability of these data to protect patient confidentiality. De-identified data may be made available upon reasonable request to the corresponding author and subject to approval by the relevant institutional ethics review board and Sahlgrenska University Hospital’s internal data access procedures.
