# Supplementary material for: Thermal intervention improves pulse oximetry accuracy in critically ill patients with low perfusion: a quasi-experimental study
Source: J Intensive Care. 2026 Jan 9;14:8. doi: 10.1186/s40560-026-00847-w (PMC12849173; doi:10.1186/s40560-026-00847-w)
Supplement: Supplementary file 1 — Additional file1 (DOCX 468 kb) [file 40560_2026_847_MOESM1_ESM.docx]

**Supplementary file.**

Thermal Intervention Improves Pulse Oximetry Accuracy in Critically Ill Patients with Low Perfusion:

A Quasi-experimental Study

Natalie Karlsson ICCRN M.Sc^a^, Felicia Olsson Lindstrand ICCRN M.Sc^b^, , Lotta Johansson ICCRN, Ph.D^b,c^, Carl Sjödin ICCRN M.Sc^b,c^*

^a^ Region Västra Götaland, Sahlgrenska University Hospital, Department of Thoracic Anaesthesiology and Intensive Care, Gothenburg, Sweden

^b^ Region Västra Götaland, Sahlgrenska University Hospital, Department of Anaesthesiology and Intensive Care, Gothenburg, Sweden

^c^  Institute of Health and Care Sciences at the Sahlgrenska Academy, University of Gothenburg, Gothenburg, Sweden

*Corresponding author at: Region Västra Götaland, Sahlgrenska University Hospital, Department of Anaesthesiology and Intensive Care, Gothenburg, Sweden, *carl.sjodin*@*gu*.se


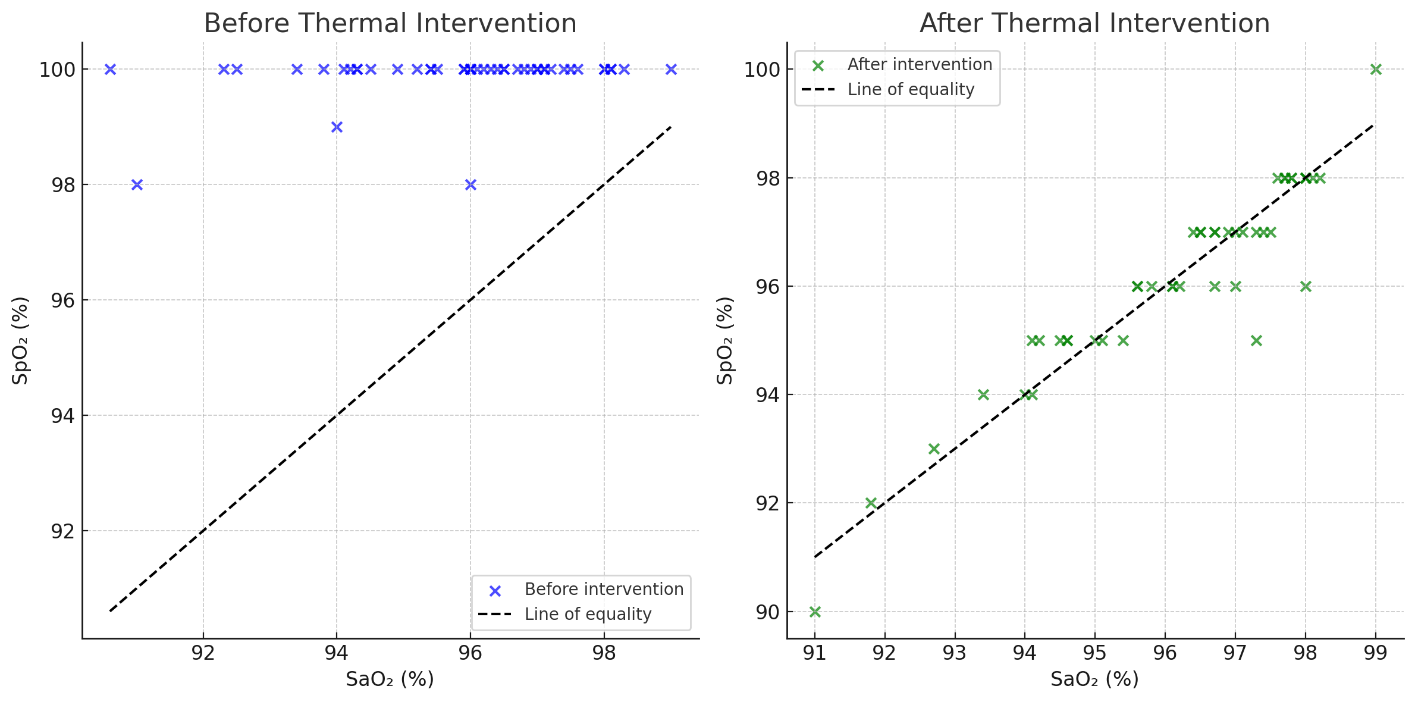


**Figure S1.** Scatterplots of pulse oximetry saturation (SpO₂) versus arterial oxygen saturation (SaO₂) before (left) and after (right) thermal intervention in 46 ICU patients with baseline perfusion index <1.0. The dashed line represents the line of equality (perfect agreement). Agreement improved substantially after warming, with R² increasing from 0.09 pre-intervention to 0.88 post-intervention.


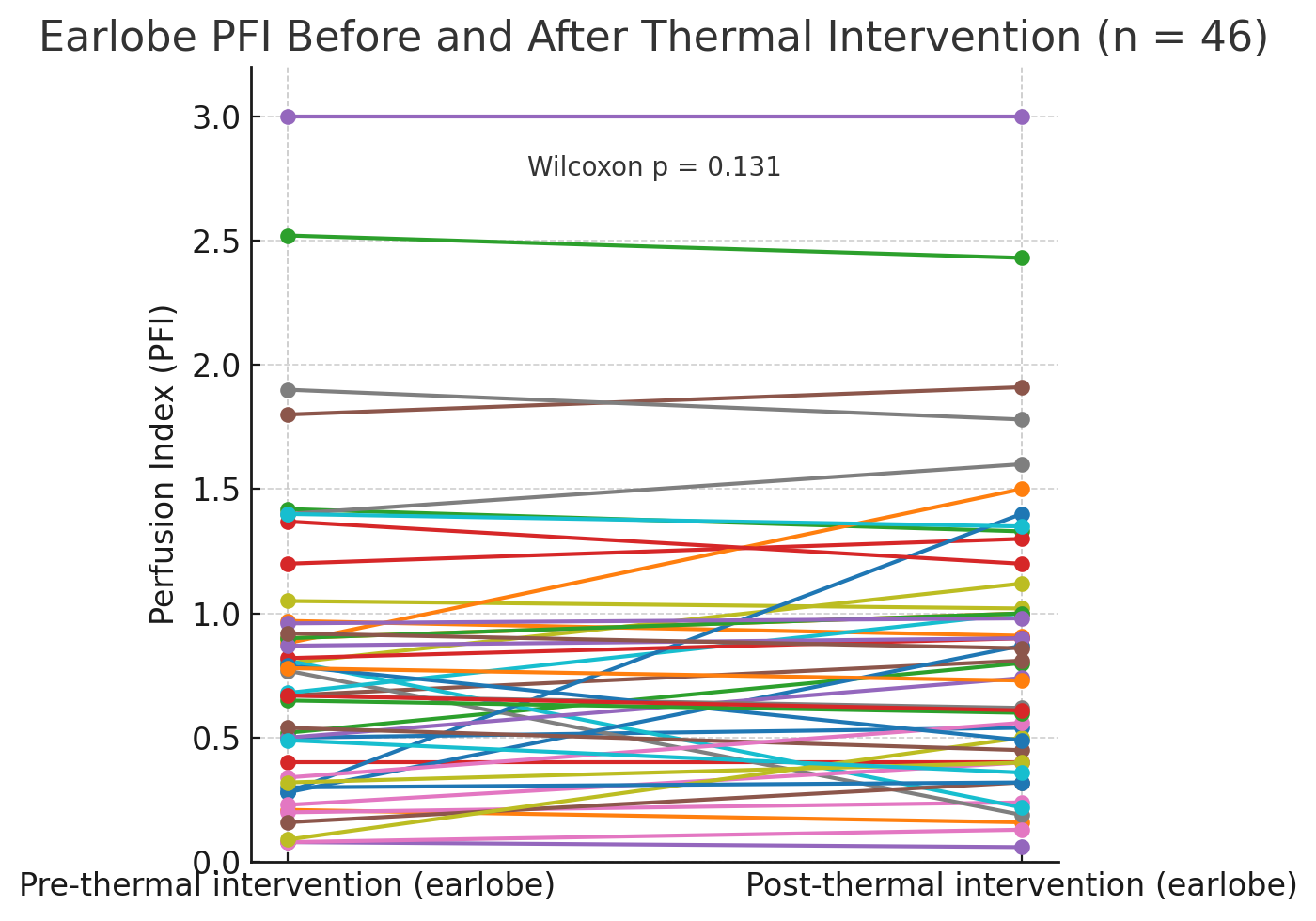


**Figure S2.** Perfusion Index (PFI) measured at the earlobe before and after thermal intervention (n = 46). Each line represents one patient. No significant change was observed (Wilcoxon signed-rank test, p = 0.131), indicating that the improvement in finger PFI was attributable to the warming intervention rather than systemic perfusion changes.


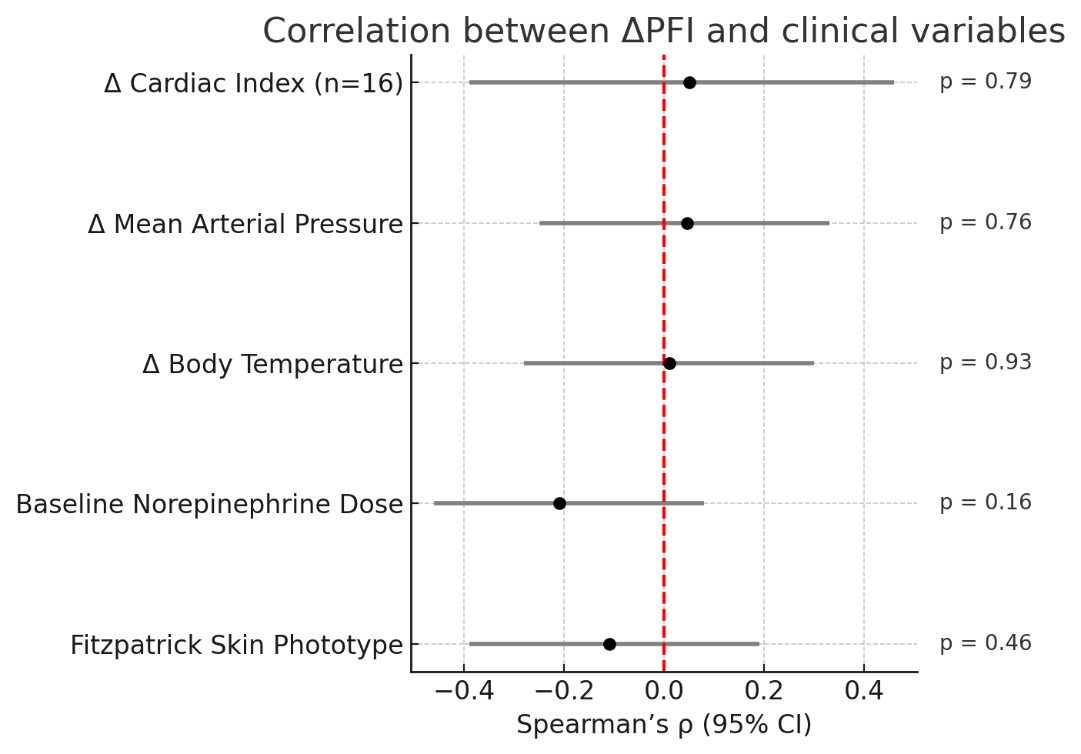


**Figure S3.** Forest plot showing Spearman’s rank correlation coefficients (ρ) with 95% confidence intervals for the association between change in perfusion index (ΔPFI) and selected clinical variables. Δ indicates post-intervention minus pre-intervention values. Positive coefficients indicate that higher values of the variable were associated with greater increases in PFI. The vertical dashed line represents no correlation (ρ = 0). p-values are shown for each correlation; none reached statistical significance at p < 0.05. CI = confidence interval; MAP = mean arterial pressure.


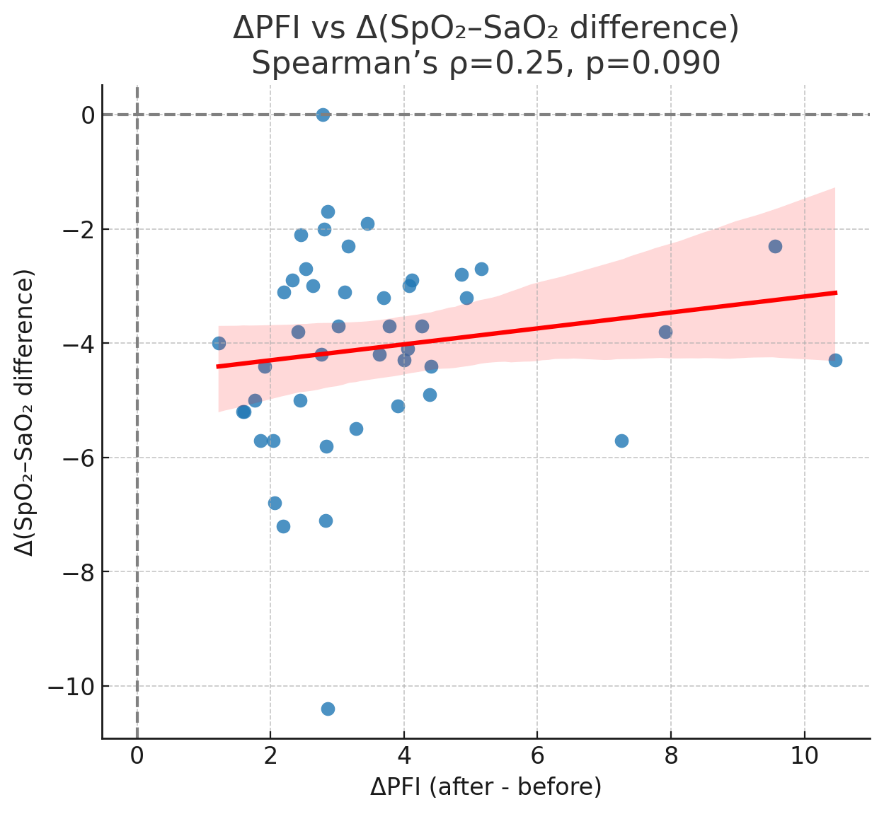


**Figure S4.** Scatterplot showing the association between change in perfusion index (ΔPFI) and change in SpO₂–SaO₂ difference after thermal intervention (n = 46). Each dot represents one patient. The red line indicates the linear regression fit with 95% confidence interval (shaded area). Spearman’s correlation coefficient was ρ = 0.25, p = 0.090, indicating a weak, non-significant positive association between ΔPFI and reduction in SpO₂–SaO₂ discrepancy.

**TREND Statement Checklist (The Role of Thermal Intervention in Optimizing Pulse Oximetry Reliability and SpO2-SaO2 Discrepancies in Critically Ill Patients - A Quasi-experimental Study)**

| TREND Item | Page(s) | Notes |
| --- | --- | --- |
| Title and Abstract: Information on how units were allocated to interventions | p.1 (Title), p.2 (Abstract) | Title specifies study type; abstract summarises design, intervention, outcomes. |
| Background: Scientific background and explanation of rationale | p.3-4 (Introduction) | Provides rationale linking low PFI to poor SpO₂ accuracy; cites relevant literature. |
| Participants: Eligibility criteria for participants, settings, and locations | p.5 (Methods - Participants) | States inclusion/exclusion criteria, ICU setting in Sweden. |
| Interventions: Precise details of the interventions intended for each group and how and when they were actually administered | p.6 (Methods - Intervention) | Describes LectroDerm warming pad, temperature, duration, and placement; control site described. |
| Objectives: Specific objectives and hypotheses | p.4 (Introduction - objectives), p.5 (Methods) | Primary aim: determine if peripheral warming increases PFI and improves SpO₂–SaO₂ agreement. |
| Outcomes: Clearly defined primary and secondary outcome measures | p.5-6 (Methods - Outcomes) | Primary outcome: change in PFI; secondary: change in SpO₂–SaO₂ agreement. |
| Sample size: How sample size was determined and, when applicable, explanation of any interim analyses and stopping rules | p.6 (Methods - Sample size) | Sample size based on pilot data for large effect size detection. |
| Assignment method: Unit of assignment, method used to assign units to study conditions, and details of any restriction | p.5 (Methods - Design: pre-post) | Pre–post design, each patient is own control; no randomisation. |
| Blinding (masking): Whether or not participants, those administering the interventions, and those assessing the outcomes were blinded to study condition assignment | Not blinded; p.5 (Methods) | No blinding of participants or assessors; justified by nature of intervention. |
| Statistical methods: Statistical methods used to compare study groups for primary outcome(s); methods for additional analyses | p.7 (Statistical analysis) | Wilcoxon signed-rank, Bland–Altman, Spearman correlations; effect sizes calculated. |
| Participant flow: Flow of participants through each stage of the study (a diagram is strongly recommended) | p.7 (Results - Flow diagram) | Flow chart in Figure 1 shows screened, excluded, included patients. |
| Recruitment: Dates defining the periods of recruitment and follow-up | p.5 (Recruitment period) | Recruitment Jan–Sep 2024; no long-term follow-up. |
| Baseline data: Baseline demographic and clinical characteristics of participants | p.8 (Table 1) | Table 1 summarises demographics and baseline physiology. |
| Numbers analysed: Number of participants included in each analysis and whether the analysis was by 'intention-to-treat' | p.7-10 (Results) | N=46 in main analysis; cardiac index subset n=16; per-protocol analysis. |
| Outcomes and estimation: Summary of results for each outcome, including effect size and confidence interval | p.9 (Results - Outcomes) | Marked PFI increase (g=2.43), bias reduction from 4.09% to 0.00%, LOA within ±2%. |
| Ancillary analyses: Summary of other analyses performed, including subgroup analyses and adjusted analyses, indicating which were prespecified | p.10 (Results - No sig. correlations) | Explored correlations with haemodynamics, norepinephrine, skin tone; none significant. |
| Adverse events: Summary of all important adverse events or unintended effects in each study group | p.11 (Safety) | No adverse events observed. |
| Interpretation: Interpretation of results, taking into account study hypotheses, sources of potential bias, and limitations | p.12-13 (Discussion) | Interprets results as clinically relevant; notes no systemic haemodynamic changes; mechanism local. |
| Generalizability: Generalizability (external validity) of the trial findings | p.14 (Generalisability section) | Suggests applicability to perioperative and other settings; cautions on extrapolation. |
| Overall evidence: General interpretation of the results in the context of current evidence | p.14-15 (Conclusion, refs) | Findings consistent with prior physiological studies; first ICU-specific evidence. |
